# The use of ultrasonography as an effective screening tool for chronic posterior cruciate ligament injuries

**DOI:** 10.1007/s10396-023-01366-z

**Published:** 2023-09-23

**Authors:** Mitsuhiro Kimura, Junsuke Nakase, Rikuto Yoshimizu, Tomoyuki Kanayama, Yusuke Yanatori, Hiroyuki Tsuchiya

**Affiliations:** 1 Department of Orthopedic Surgery, Graduate School of Medical Science, Kanazawa University, 13-1 Takaramachi, Kanazawa, 920-0934 Japan; 2 Department of Orthopedic Surgery, National Hospital Organization, Kanazawa Medical Center, Kanazawa, Japan

**Keywords:** Ultrasonography, Chronic, Posterior cruciate ligament, Diagnosis

## Abstract

**Purpose:**

We aimed to explore the applicability and validity of ultrasonography for diagnosing chronic posterior cruciate ligament (PCL) injuries.

**Methods:**

PCL thickness was measured at 2 cm proximal to the tibia insertion site. Using the same ultrasonography image, the angle tangent to the PCL from the tibia insertion site was also measured. These data were analyzed by plotting the receiver operating curve (ROC), and the sensitivity and specificity were calculated according to the optimal cut-off point. Ultrasonography data from the PCLinjured knee were compared with those from the contralateral uninjured knee of the same patient.

**Results:**

Twelve men and six women, with a mean age of 28.8 ± 14.0 years, were included in this study. The mean time from injury to medical examination was 10.0 ± 6.7 months. The mean thickness of the PCL was 8.1 ± 1.9 mm on the affected side and 5.8 ± 1.2 mm on the uninjured side, with the affected side being significantly thicker. ROC analysis revealed that the optimal cut-off value for the thickness of chronic PCL injuries was 6.5 mm (sensitivity 83.3%, specificity 77.8%, area under the curve [AUC] = 0.87). The optimal cut-off value for the angle was 20° (sensitivity 88.9%, specificity 94.4%, AUC = 0.96).

**Conclusion:**

Ultrasonography is useful as a screening tool for chronic PCL injuries. The optimal cut-off point was 6.5 mm for thickness and 20° for angle.

**Level of evidence:**

IV.

## Introduction

The posterior cruciate ligament (PCL) is the toughest ligament in the knee and is recognized as an important stabilizer [1]. PCL injury can cause pain, swelling, and instability in the short term, limiting participation in sports. Additionally, symptomatic PCL injuries left untreated increase the occurrence of developing osteoarthritis (OA) of the knee over the long term [2–5]. Consequently, approximately 80% of patients with chronic PCL deficiency suffer irreversible cartilage damage [5]; hence, a timely and accurate diagnosis of PCL injury is necessary to prevent the development of post-traumatic OA. However, this injury is often missed and the actual incidence rate is unknown, with the reported incidence of PCL injuries in the literature varying widely from 4 to 38% of all knee traumas [6, 7].

**Fig. 1:**
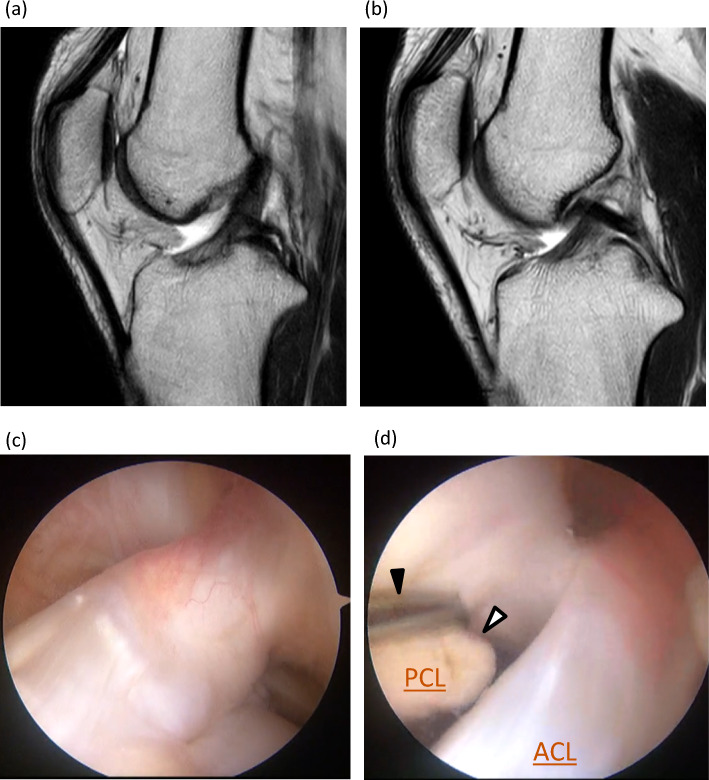
A representative case of chronic PCL injury; a 26-year-old man, injured 3 years ago when he sprained his left knee while snowboarding. **a** Sagittal MRI image of the left knee; the ACL appears to be injured. **b** There is continuity in the PCL. **c** Arthroscopic findings; the ACL was deflected, but when the tibia was pulled forward, tension was normal and there was no injury. **d** Probing PCL; PCL was partially injured. The black arrowhead represents the tip of the probe, and the white arrowhead represents the torn end of the PCL from the femoral side. ACL, anterior cruciate ligament; PCL, posterior cruciate ligament; MRI, magnetic resonance imaging

Imaging evaluation methods for PCL injuries include magnetic resonance imaging (MRI) and radiographs with posterior stress. MRI is the "gold standard" in imaging, with an uninjured intact PCL appearing as a well-defined continuous zone of low signal intensity on MRI images [8]. Injured ligaments show swelling, hematoma in the surrounding area, disruption of continuity, and signal changes within the ligament. The sensitivity of detecting acute (< 6 weeks) PCL injury is reported to be 96%–100% [9–11]. In contrast, for chronic (≥ 6 weeks) PCL injuries, the sensitivity is as low as 62.5%, making it relatively difficult to diagnose PCL injuries during this phase [9] (Fig. 1). MRI is also costly, requires long examination times, and is contraindicated in patients with pacemakers, magnetic metal implants, and claustrophobia [12].

Ultrasonography has the advantages of low cost, short examination times, no contraindications, and no radiation exposure [12, 13]. It is widely used to diagnose ligament, tendon, and meniscus injuries and is a good modality for screening intra-articular lesions [12, 14, 15]. Some studies using ultrasonography have shown that the injured PCL is thicker than the contralateral non-injured PCL or the PCL of healthy people [16, 17]. Wang et al. recommended a thickness of ≥ 6.5 mm based on ultrasonography measurement as the diagnostic criteria for acute PCL injury (sensitivity 90.6%, specificity 86.7%) [18]. However, there are no reports on the cut-off point for diagnosing chronic PCL injuries. Moreover, chronic PCL injuries are often difficult to diagnose, even with MRI, and it is important to increase the diagnostic yield during this phase.

In this study, we aimed to explore the applicability and validity of ultrasonography for diagnosing chronic PCL injuries. We hypothesized that ultrasonography would be an effective screening tool for chronic PCL injuries in the acute phase.

## Materials and methods

### Patients

This study was performed in accordance with the ethical standards as laid down in the 1964 Declaration of Helsinki and its later amendments or comparable ethical standards. The study design was approved by the Ethics Committee of Kanazawa University Hospital (approval no. 1842). All patients were informed about the study purpose and procedures, and informed consent was obtained from all patients.

We included 18 patients with PCL injuries who visited our outpatient clinic between April 2020 and March 2022. The inclusion criteria were (1) age ≥ 18 years; (2) time from injury to examination ≥ 3 months; and (3) those with who have at least one of the following clinical findings: (a) positive posterior drawer test, (b) positive for posterior sagging sign, and (c) positive dial test at both 30° and 90° knee flexion. The following patients were excluded: (1) patients with a history of PCL reconstruction, (2) patients with multiple ligament injuries, (3) patients with PCL tibial attachment avulsion fracture, and (4) patients with a history of trauma such as fracture in either knee. Ultrasound data from the PCL-injured knee were compared with those from the contralateral uninjured knee of the same patient.

### Ultrasonographic evaluation

**Fig. 2:**
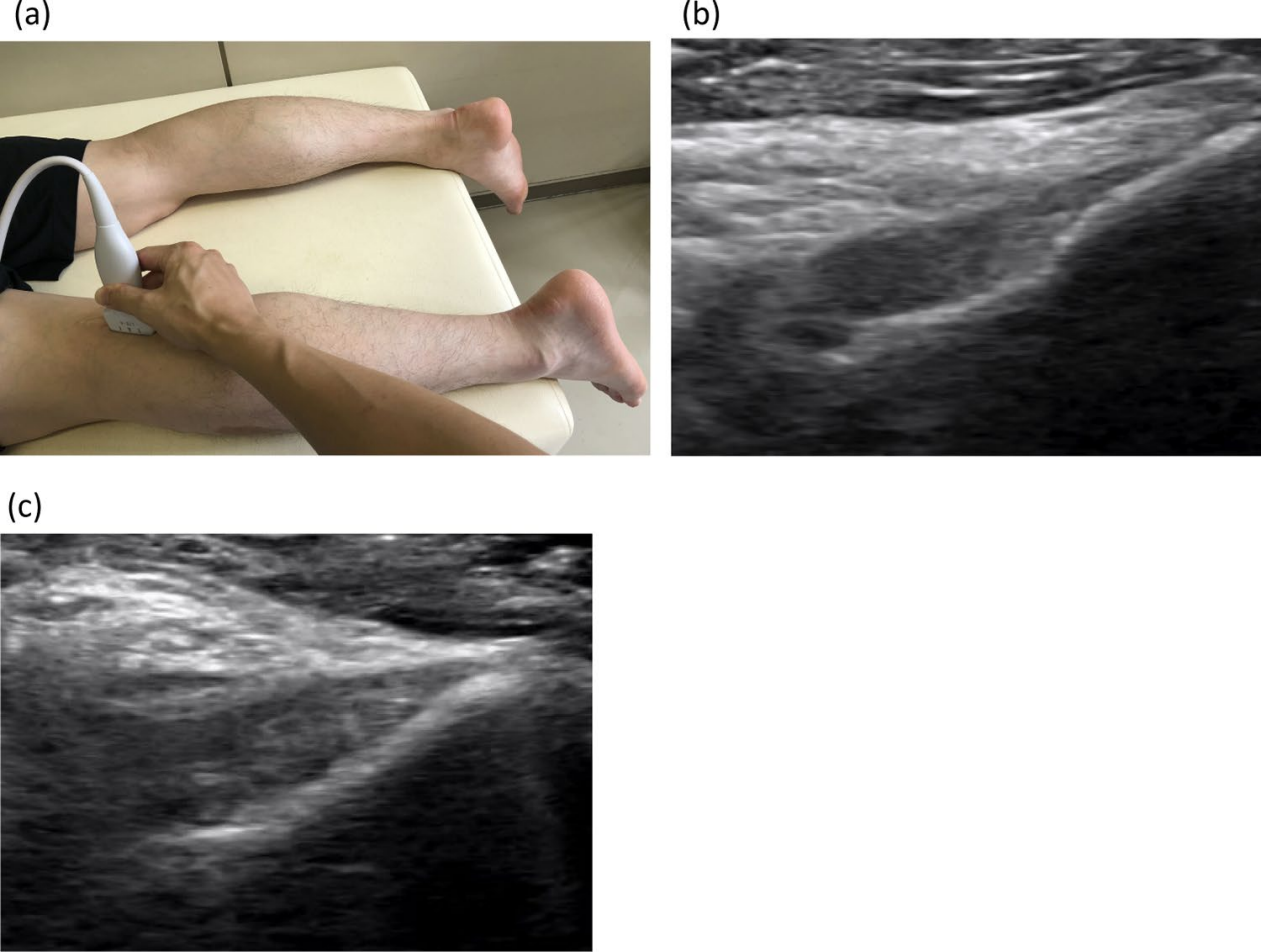
Ultrasonographic delineation of the posterior cruciate ligament. **a** The patient was placed in the supine position with the knee in full extension, and the probe was placed in the popliteal region, longitudinally from the intercondylar area of the proximal tibia. **b** An ultrasound image of the same patient as in Fig. 1; on the image of the healthy side, the posterior cruciate ligament is depicted as a low-echoing fan-shaped structure tapering distally in the proximal tibia. **c** Ultrasound image of the affected side showing a thicker posterior cruciate ligament compared to the healthy side

**Fig. 3:**
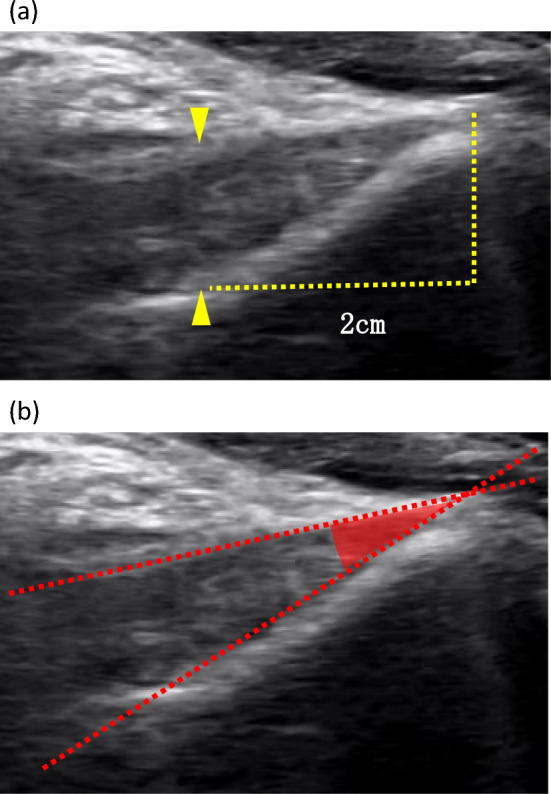
Ultrasonographic measurement method of the posterior cruciate ligament. **a** The thickness of the posterior cruciate ligament was measured at 2 cm proximal to its insertion into the tibia (between the yellow arrowheads). **b** The angle of the posterior cruciate ligament was measured using a tangent line drawn from the tibial insertion

Ultrasonographic measurements were performed using SNiBLE (KONICA MINOLTA, Tokyo, Japan) with an 18-MHz linear probe. The patient was placed in the prone position with the knee in full extension, and the transducer was placed in the popliteal region, longitudinally from the intercondylar area of the proximal tibia. First, the posterior tibial plateau was located, and then the transducer was rotated in an oblique sagittal plane to visualize the fibrillar band-like structure extending toward the posterior medial femoral condyle. The PCL is a fan-shaped structure, thick proximally and tapering distally (Fig. 2) [16]. We used the image with the thickest PCL for our measurements. The thickness of the PCL was measured at the site 2 cm proximal to the insertion site into the tibia based on previous ultrasound studies for acute PCL injuries [18]. Using the same ultrasonographic image, the angle tangent to the PCL from the tibia insertion site was also measured (Fig. 3). The accuracy of thickness and angle was 0.01 mm and 0.01° respectively, after rounding to 1 decimal.

Ultrasonographic measurements were performed by two experienced musculoskeletal ultrasound physicians (M.K. and R.Y.) to determine inter-observer reliability using the intraclass correlation coefficient (ICC). The two examiners who performed the ultrasonographic measurements were independent and were not directly involved in the patient's treatment. Thus, the evaluator could not distinguish the PCL-injured knee at the time of the ultrasonographic measurement. Each observer repeated measurements at 4-week intervals to determine intra-observer reliability.

### Clinical evaluation

Posterior instability was evaluated as the amount of total anterior–posterior translation with maximum manual withdrawal and pushing. These values were measured with the participant in the supine position and knee flexion at 70^o^ using a KT-1000 arthrometer (MEDmetric, San Diego, California, USA), and differences versus the uninjured side were recorded to the nearest 1 mm [19, 20]. The International Knee Documentation Committee (IKDC) score was also examined.

### Statistical analysis

All measurements were analyzed using SPSS v24.0 (IBM Corp, Armonk, NY, USA). The statistical data were expressed as mean ± standard deviation (SD). The PCL thickness and angle were compared with those of the uninjured side using paired t-test. A receiver operating curve (ROC) was plotted and analyzed for PCL thickness and angle, and sensitivity and specificity were calculated according to the optimal cut-off point. Pearson's or Spearman's correlation coefficients were used to calculate the correlation of angle, anteroposterior displacement, and IKDC score to PCL thickness, respectively. *p* < 0.05 was set as the significance level.

The recommended sample sizes were evaluated by computing statistical power using G-Power 3.1 software (Heinrich-Heine University Dusseldorf, Dusseldorf, Germany), based on a previous study [18]. A minimum of 17 samples per group (effect size 0.80, alpha error 0.05, and target power 0.8) were recommended, as in previous studies.

## Results

**Fig. 4:**
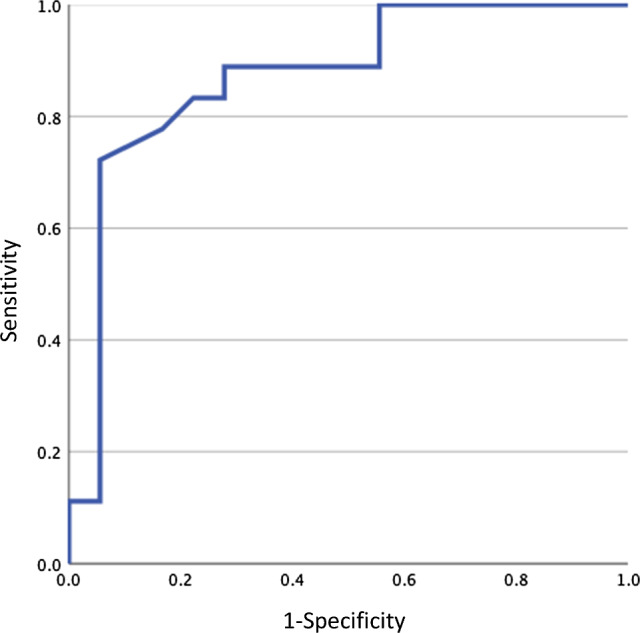
The result of the receiver operating characteristic curve analysis for chronic posterior cruciate ligament injury diagnosis regarding the degree of thickness

**Fig. 5:**
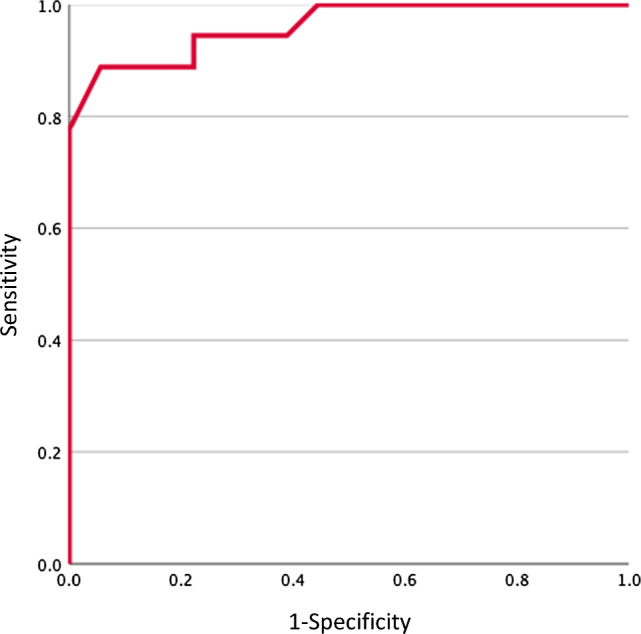
The result of the receiver operating characteristic curve analysis for chronic posterior cruciate ligament injury diagnosis regarding the extent of the angle

Twelve men and six women, with a mean age of 28.8 ± 14.0 years, were included in this study. The mean time from injury to medical examination was 10.0 ± 6.7 months. Four patients underwent PCL reconstruction. The mean PCL thickness was 8.1 ± 1.9 mm on the affected side and 5.8 ± 1.2 mm on the uninjured side, with the affected side being significantly thicker. The optimal cut-off points for each are shown in Table 1. ROC analysis revealed that the optimal cut-off value for the thickness of chronic PCL injuries was 6.5 mm (sensitivity 83.3%, specificity 77.8%, area under the curve [AUC] = 0.87) (Fig. 4). The optimal cut-off value for the angle was 20° (sensitivity 88.9%, specificity 94.4%, AUC = 0.96) (Fig. 5). When the PCL thickness was ≥ 6.5 mm and/or the PCL angle was ≥ 20°, the sensitivity and specificity for detecting chronic PCL injury were 94.4% and 66.7%, respectively.

**Table 1:** ROC curve analysis result for evaluation of the diagnostic ability for chronic PCL injury

	Thickness (mm)	Angle (°)
Cut-off value	6.5	20
Sensitivity (%)	83.3	83.9
Specificity (%)	77.8	94.4
AUC	0.87	0.96
95% CI	0.749–0.995	0.905–1

AUC, area under the curve; CI, confidence interval; PCL, posterior cruciate ligament; ROC, receiver operating characteristic

A significant positive correlation was observed between PCL thickness and angle (r = 0.69, *p* < 0.01). Conversely, no correlation was identified between PCL thickness and the amount of anteroposterior translation or IKDC score (*p* = 0.80 and 0.25, respectively). Intra- and inter-observer reliability obtained using the ICC for PCL thickness and angle measurements were 0.968 and 0.876 and 0.939 and 0.868, respectively.

## Discussion

The most important finding of this study was the usefulness of ultrasound as a screening examination for patients with suspected chronic PCL injuries. Previous studies have used ultrasound and demonstrated its effectiveness in diagnosing acute PCL injuries; however, none has investigated chronic injuries. This study revealed that the mean thickness of the injured PCL and the angle of the PCL from the tibial attachment site were significantly greater than those of the uninjured, healthy PCL, as was the case in the acute phase, even in the chronic state, at least 3 months after the injury. The sensitivity and specificity were 83.3% and 77.8%, respectively, for a PCL thickness ≥ 6.5 mm as a diagnostic criterion. When the angle was ≥ 20°, the sensitivity and specificity were 83.3% and 94.4%, respectively (both good results). When these were combined, i.e., when at least one of these two diagnostic criteria was positive, the sensitivity was even higher (sensitivity: 94.4%, specificity: 66.7%). From the results of this study, in patients with residual knee instability after a lower leg contusion or other injury, if a chronic PCL injury is suspected based on physical findings such as a positive posterior sagging sign, diagnosis can be made in most cases by first measuring the PCL thickness and the angle tangent to the PCL from the tibia insertion site with ultrasound, rather than using radiographs with posterior stress or MRI.

When the PCL is injured, the pattern of tissue destruction is variable. In complete tears on MRI images, the ligament fibers are often discontinuous, with signal changes completely crossing the ligament fibers. In partial tears, the PCL is often high-signal and thickened [10]. Reportedly, the PCL can heal so that signal changes gradually disappear and fiber continuity can be regained [3, 10, 21]. According to Rodriguez et al., the mean time from injury to MRI was 54.8 days (range: 1–529 days), confirming that the PCL was continuous in 62% of cases [10]; however, the PCL may heal in an elongated state due to gravity and persistent posterior tibial subluxation during the recovery process after injury when the injured ligament is continuously scarred and appears intact [4, 5]. Therefore, several authors reported that even in MRI, the appearance of the PCL is not reliable in predicting its functional integrity or degenerative changes [10, 21–23].

In previous attempts to improve the diagnostic rate of patients with chronic PCL injuries, Wilson et al. quantified transverse relaxation time (T2) mapping values at 3 Tesla for six patients with chronic PCL injuries in whom fibers appeared continuous on conventional MRI images, and reported that the mean T2 values for patients with chronic PCL injuries were higher than those reported for asymptomatic volunteers [24]. Kim et al. compared both supine lateral radiographs at 90° knee flexion (90 SLRs) and Telos stress radiographs in patients with chronic PCL injuries (n = 38). The results showed that the difference between injured and normal knees was significantly greater in patients with chronic PCL injuries compared to healthy controls (7.5 ± 3.5 mm vs. 1.2 ± 1.0 mm, *p* < 0.001), indicating that the 90 SLRs are reliable alternative methods for assessing the posterior knee laxity when stress radiographs are not available [25].

Ultrasound has many advantages over these examination methods, including time and cost savings, simplicity with no special equipment, and ease of comparison with the contralateral side. In addition, the dynamic and continuous delineation of ligament fibers allows measurements with a true sagittal image, which would be an appropriate screening tool. The angular measurements, in particular, were more sensitive and specific. Compared to thickness measurements, the results were less affected by individual differences in age, sex, height, and body weight, which may be superior in terms of diagnostic efficiency.

This study had several limitations. First, it was conducted at only one medical institution, and the sample size was small. Therefore, a multicenter study with a larger number of cases is needed to validate this diagnostic criterion. Second, the accuracy of ultrasonography is highly dependent on the examiner's experience, with a certain degree of experience being necessary to achieve satisfactory diagnostic accuracy. Third, MRI served as the "gold standard" for PCL injuries, and arthroscopic confirmation was not performed except in surgical cases. Fourth, this was a cross-sectional study in which measurements were recorded only at one time point during the chronic phase after 3 months of injury, and more detailed longitudinal studies are needed to determine how the injured PCL changes from the acute to the chronic phase. Fifth, although PCL thickness is likely to be affected by physique, we did not investigate the difference or ratio of measurements between the uninjured and affected sides. For simplicity with fewer measurements, we determined the diagnostic criteria based on the measurement of only the affected side but still obtained a high sensitivity and specificity. Finally, PCL thickness did not correlate with posterior instability as measured by the KT-1000 or with clinical performance as measured by the IKDC score. In other words, the degree of posterior laxity did not completely correlate with PCL morphological changes on ultrasound. It is unclear which patients will ultimately require surgery, and other investigations are needed. However, this is the first study to demonstrate the usefulness of ultrasonography for diagnosing chronic PCL injuries, which are often missed.

## Conclusion

Ultrasonography is useful as a screening tool for chronic PCL injuries. The optimal cut-off points were a thickness of 6.5 mm and an angle of 20°.

## Data Availability

The data that support the findings of this study are available on request from the corresponding author, MK. The data are not publicly available due to their containing information that could compromise the privacy of research participants.
